# Trust in Healthcare Providers Among American Indians in the Midwest

**DOI:** 10.3390/ijerph23030404

**Published:** 2026-03-23

**Authors:** Laura Porto-Roquett, Dasy Resendiz, Ryan Goeckner, Joseph Pacheco, Sean M. Daley, Won S. Choi, Christine Makosky Daley

**Affiliations:** 1 Institute of Indigenous Studies, Lehigh University, Bethlehem, PA 18015, USA; lmp222@lehigh.edu (L.P.-R.);; 2 Department of Community & Global Health, College of Health, Lehigh University, Bethlehem, PA 18015, USA; 3 Department of Population Health, College of Health, Lehigh University, Bethlehem, PA 18015, USA; 4 Department of Biostatistics and Health Data Science, College of Health, Lehigh University, Bethlehem, PA 18015, USA

**Keywords:** American Indians, medical mistrust, prescription drug misuse, continuity of care

## Abstract

**Highlights:**

**Public health relevance—How does this work relate to a public health issue?**

- This research addresses the critical public health issue of prescription drug misuse, which affected approximately 14.4 million Americans in 2023.
- The study identifies a statistically significant association between low trust in healthcare providers and the misuse of prescription medications among American Indians.

**Public health significance—Why is this work of significance to public health?**

- It highlights that misuse, including sharing medications or not finishing antibiotic courses, leads to severe community health problems such as antibiotic resistance.
- Practitioners and policymakers should focus on increasing continuity of care, as having a personal doctor is positively related to higher trust and better health outcomes.

**Public health implications—What are the key implications or messages for practitioners, policy makers and/or researchers in public health?**

- Researchers and administrators need to address high staff turnover and vacancy rates in the Indian Health Service (IHS) and tribal clinics, as these structural issues currently prevent the long-term relationships necessary to build patient trust.

**Abstract:**

Prescription drug misuse disproportionately impacts American Indian communities, yet limited research explores how trust in healthcare settings affects behaviors related to prescription drug use. Using data from a 2017 cross-sectional survey of 781 American Indian adults in the Plains region, this study aims to examine the association between trust in health information provided by physicians and the misuse of prescribed medication, while identifying demographic and structural factors that influence trust levels. To assess trust, the study utilized a tool consisting of questions adapted from the Health Information National Trends (HINTS) survey, which asked respondents to rate how much they trust health and medical information from their doctors. Results showed that 29.3% of participants reported high trust in provider information. Trust was significantly higher among women, individuals with private insurance, and those with a personal healthcare provider. Notably, participants who misused prescription drugs reported significantly lower trust (30.0%) than those who did not (40.0%). The study concludes that while historical trauma influences mistrust, structural factors like continuity of care and regular provider access are vital. Improving patient–provider relationships may reduce medication misuse and associated risks like antibiotic resistance.

## 1. Introduction

Prescription drug misuse is a public health problem in the United States (US) [1]. According to the National Survey on Drug Use and Health, in 2023, about 14.4 million Americans aged 12 or older misused prescribed medication, specifically psychotherapeutics, including pain relievers, tranquilizers/sedatives, or stimulants [2]. American Indians and Alaska Natives misuse prescription pain relievers more than other racial or ethnic groups, with 5.7% of American Indians and Alaska Natives misusing them compared to 3.0% of Whites and 3.7% of African Americans. Similar disparate percentages are seen in opioid and Central Nervous System (CNS) stimulant misuse [2]. While pain relievers represent a critical area of concern, the scope of this study encompasses the misuse of various prescription medications, including antibiotics and stimulants, to explore how trust influences diverse medication-taking behaviors.

Misuse of prescription medication occurs when an individual takes the medication for a shorter or longer period of time than directed. It also includes when a prescription drug is taken by an individual who is not the intended patient, regardless of whether the user is using it to treat a legitimate medical complaint or to get high [3]. Besides the misuse of pain relievers, CNS stimulants, tranquilizers, and sedative medications, there is also misuse of other types of prescription medication, such as antibiotics, which are never used for recreational purposes or due to addiction. The misuse of antibiotics occurs through sharing or not finishing a course when prescribed, which creates problems such as antibiotic resistance [4].

Despite higher rates of prescription drug misuse among American Indians and Alaska Natives, research exploring this misuse and the reasons behind it is sparse. We first examined prescription medication sharing among adult American Indians in the Plains region in 2011 and found that approximately 30% of participants had taken medication prescribed for someone else. The most commonly misused prescription medications were pain relievers (23.6%) and antibiotics (22.0%). These results, particularly the high rates of misuse of antibiotics, show a lack of use or access to appropriate medical care, rather than using prescription medications for recreational purposes or addiction. This highlights the possibility that participants are unwilling to seek medical care for a variety of reasons, including mistrust of healthcare providers [5], an important barrier to seeking care for American Indians and Alaska Natives due to historical trauma [6].

Trust is vital to public confidence in health and science [7]. Trust in a physician means that the patient is willing to be vulnerable, believing the physician will act in their best interest [8]. This definition aligns with the Integrative Model of Organizational Trust, which posits that trust is built on perceived benevolence and integrity [9]. Research exploring patients’ trust shows higher patient satisfaction and greater continuity of care. It also shows an association between trust and adherence to treatment plans, self-reported health improvement, and increased ability to manage chronic diseases [5].

Research exploring the association between historical trauma and substance use disorder among American Indians and Alaska Natives has been conducted previously [10,11,12,13], highlighting that historical unresolved grief contributes to or exacerbates the current social pathology of trauma and alcoholism, substance misuse, and other social problems in American Indian and Alaska Native communities [14]. Furthermore, medication misuse is also associated with other factors. For example, research shows that misuse, particularly of antibiotics, often stems from low health literacy [15] or a lack of access to a provider for consultation [16].

A study evaluating the relationship among trust in healthcare providers, prior healthcare experiences, and structural characteristics of healthcare in a national sample of African Americans and Whites shows that African Americans are more likely to report low trust in healthcare providers. This study suggests that experiences with health care providers and sources of medical care may be more important to trust in health care providers among African Americans than sociodemographic factors [17]. Studies about trust in health care providers among American Indians and Alaska Natives are sparse. However, a study to identify barriers and facilitators to good clinical interaction in an Indian Health Service (IHS) hospital found trust as a central theme in the determination of whether an interaction is considered good or bad [18].

A study to provide insight into the healthcare experiences of American Indian women found, among other barriers, that negative provider relationships, including rushed or rude provider interactions, the provider not listening or ignoring patient concerns, poor, inaccurate, or inadequate care or diagnosis, discrimination in healthcare, and the need for personal relationships with providers, to be important structural issues that contribute to health disparities [19]. Furthermore, research among American Indians and Alaska Natives exploring trust in health care providers and their association with prescription drug misuse is sparse.

While existing research has established high overall rates of substance use disorders in American Indian and Alaska Native communities, there is a significant lack of data regarding the relationship between medical trust and prescription drug behaviors. Specifically, it is not well understood how mistrust influences misuse. Understanding this link is relevant because prescription drug misuse can lead to important public health problems, including antibiotic resistance and increased mortality.

The primary objective of this research is to examine the association between trust in health information provided by physicians and the misuse of prescribed medications, and to identify specific demographic and structural factors, such as gender, insurance status, and continuity of care, that influence trust levels within American Indians from the northern plains.

The scope of this study is focused on 781 American Indian adults residing in both urban and reservation communities. The findings are limited by the focus on a specific geographic region, which may not represent all American Indian nations. Additionally, the cross-sectional nature of the data prevents the establishment of a causal link between trust and misuse. Furthermore, all data are self-reported, which may be subject to recall or response bias.

This paper is a secondary analysis of the data from a study on mental health, addiction, and prescription drug misuse among American Indians in the Plains region in 2017, conducted by American Indian Health Research and Education Alliance (AIHREA) researchers, using a 139-item instrument, which includes a series of questions to explore trust in health information given by healthcare providers. The original study employed a community-based participatory research approach from survey inception to dissemination.

## 2. Materials and Methods

A cross-sectional survey was conducted in 2017 by investigators from Lehigh University with affiliations with the American Indian Health Research and Education Alliance (AIHREA) [20]. Participants came from both urban and reservation communities in the Plains region of the United States, focused on mental health, addiction, and prescription drug misuse, using a community-based participatory research (CBPR) approach. This approach includes community members throughout all phases of research, from concept inception through implementation, analysis, and dissemination [21]. This approach is the hallmark of AIHREA projects, whose academic research team consisted of approximately 80% American Indian researchers and students and 20% non-American Indian researchers and students. American Indian community members outside of the research team were engaged in the process throughout the study, including members of a longitudinal community advisory board who provided input on multiple studies conducted with their communities and representatives from various community partners with a specific interest in this study. Data was collected in 2017 as part of a larger community-based study. The present manuscript represents a focused secondary analysis of the original dataset. Consistent with CBPR principles, initial dissemination efforts prioritized returning findings to participating communities prior to academic publication. The behavioral health concerns examined remain persistent and highly relevant in Plains-region American Indian communities.

The survey instrument consisted of 139 questions with multiple skip patterns determined by an individual’s answers to questions about the use of various substances. Because of skip patterns, the survey was typically much shorter; the time to complete it ranged from about 15 min to 45 min, though most participants completed it in less than 30 min. Topics covered included demographic information, questions about spirituality, questions about trust in health information and providers, a series of mental health measures, perceptions of alcohol and drug use by others in a respondent’s community, perceptions of health risks associated with alcohol and drug use, and personal use of alcohol and different types of illicit and prescription drugs. Here, we report only on demographic information, select mental health measures, prescription drug misuse, and trust in health information and providers, as described below. All data were self-reported and collected both online, using the secure REDCap^TM^ program, and using paper and pencil. Paper surveys were used in circumstances where it was difficult or impossible to obtain a reasonable bandwidth for collecting data, primarily in reservation communities. A similar survey was completed with some of the participating communities in 2011 [5].

Participants were recruited at community events held by AIHREA or partner organizations and tribes, including powwows and other cultural events, health fairs, and research-focused community events, specifically a type of event we created, a community research forum. A community research forum is a one-day event that pairs a community cultural activity, most often a powwow, with research activities, including both data collection (e.g., surveys, interviews, focus groups) and dissemination of results through posters, presentations, and discussions. Though this was a convenience sample, we were able to achieve a total of 968 participants, providing us with useful information concerning addiction and mental health among American Indians in both reservation and urban locations in the Plains. Because participants were permitted to skip any questions they would prefer not to answer, the final number of participants in this sample related to trust in information about health and providers is 629. The eligibility criteria for the study included self-identification as American Indian, age 18 or older, and willingness to participate. Participants received a $20 gift card to a local store for participation; stores varied based on the recruitment event and were selected by our community advisors.

IRB approval. Appropriate institutional review board and human subject approvals were received from Tribal partners and the university’s Institutional Review Board before implementation of the survey. The study protocol titled “Understanding American Indian Mental Health and Addiction” (STUDY00140271) was reviewed and approved by the IRB (IRB00000161) in November 2016. The research was categorized as expedited behavioral research.

Measures. Demographics. Standard demographic information was collected, including age, gender, race/ethnicity, tribal affiliation, marital status, children, education level, self-reported health status, insurance status, and the type of provider seen and frequency of use. Questions were also asked about personal ties to Native identity and cultural activities. For this analysis, one question about Native identity was included: “Some people talk about living life in traditional ways. To what extent do you follow the Native way of life?” Answer choices included “Not at all,” “Rarely,” “Sometimes, and “A lot.” Finally, a series of questions about ties to Native spirituality was asked using the Native American Spirituality Scale. The Native American Spirituality Scale is an 8-item measure requesting yes or no answers and is scored by assigning one point to each item that is endorsed. A mean score is calculated and interpreted as high, moderate, or low using tertiles [22].

Mental Health Measures. Measures of anxiety, depression, perceived stress, and perceived social support were included. To assess anxiety, the Beck Anxiety Inventory (BAI) was used. The BAI consists of 21 items on a four-point scale, and scores can range from 0 to 63 [23]. Cut points used to assess anxiety include low anxiety (0–21), moderate anxiety (22–35), and concerning anxiety (36+). To assess depression, the Center for Epidemiologic Studies Depression Scale (CESD-10) was used. The CESD-10 consists of 10 items on a four-point scale, with two questions being reverse-scored, and scores can range from 0 to 30 [24]. Any score that equals 10 or above is considered depressed. To assess perceived stress, Cohen’s [24] 10-item Perceived Stress Scale (PSS-10) was used. The PSS-10 consists of 10 items on a five-point scale, with four questions being reverse-scored. and scores can range from 0 to 40 [25]. Cut points used to assess stress include low stress (0–13), moderate stress (14–26), and high perceived stress (27–40). To assess social support, the Multidimensional Scale of Perceived Social Support (MSPSS) was used. The MSPSS consists of 12 items on a seven-point scale, and mean scores can range from 1 to 7 [26]. Cut points used to assess social support include low support (1–2.9), moderate support (3–5), and high support (5.1–7).

These mental health measures have previously been used within American Indians or indigenous contexts. De Coteau et al. [27] examined the reliability and validity of the BAI in a sample of Northern Plains American Indians, finding strong internal consistency and supporting its construct validity. Schure and Goins [28] performed a psychometric evaluation of the CES-D with a sample of older American Indians, confirming the tool’s concurrent and divergent validity and found the scale to have excellent internal consistency. Gray et al. [29] assessed the multiple depression measures, including the CES-D, among 473 American Indians, reporting excellent internal consistency and demonstrating convergent validity with the BECK Depression Inventory-II. Regarding the PSS-10, Mitchell et al. [30], while the study was not exclusive to American Indian populations, the study is frequently cited in Indigenous mental health research to justify the use of the PSS-10 in diverse traumatized populations. Finally, the MSPSS was recently validated in an Indigenous context. The study by McCormick et al. [31] confirmed the original three-factor structure (family, friends, and special person) and reported excellent reliability and structural consistency, affirming its utility as a valid measure of social support in indigenous cultural frameworks.

Prescription Drug Misuse. To assess prescription drug misuse, a set of questions previously created and used in a survey in the same population, in conjunction with our community advisors, was modified and used [5]. Respondents were asked if they had ever taken medicine of any kind that was not prescribed for them, and if so, what kind. Possible answers included allergy medicine, antacids, antibiotics, asthma medicine, blood pressure medicine, cholesterol medicine, diabetes medicine, epilepsy or seizure medicine, heart medicine, pain relievers, water pills, or other (fill in). Participants were then asked how often they took medicine that had not been prescribed to them with the following answer choices: “I have only taken medicine that was not prescribed for me once,” “Once every few years,” “Once per year,” “A few times per year,” “Monthly,” “Weekly,” or “Daily.” A similar series of questions was asked about whether they had ever given someone else their prescription medication. Finally, participants were asked if they had ever not finished antibiotics that were prescribed to them to obtain specific information about the problem of antibiotic resistance.

Trust Measures. To assess trust, a set of questions was created by our team based on questions from the Health Information National Trends (HINTS) survey [32]. This tool measures the dimension of informational trust, specifically, how much respondents trust medical information from various sources. Respondents were asked how much they trust information about health and medical topics from their doctors. The answer choices were as follows: “A lot”, “Some”, “A little”, or “Not at all”. A similar series of questions was asked about whether they trust information about health and medical topics from religious leaders, tribal leaders, and family or friends. “A lot” responses in each one of these measures were considered “High trust”, “Some” and “A little” responses were regrouped as “Moderate trust”, and “Not at all” were considered as “No trust”.

Data Cleaning and Analysis. Some survey data were collected via paper and pencil rather than using direct participant entry via REDCap^TM^ (version 7.2.2) due to bandwidth problems in certain locations. These surveys were double-entered into identical REDCap^TM^ databases by research staff members, then compared and cleaned to ensure fidelity to the raw data. Cleaned data entered from the paper surveys were combined with participant direct-entry data into one CSV file for analysis. Analysis was performed in IBM SPSS^TM^ (version 31).

Frequency counts and percentages of the demographic variables were calculated for individuals for the trust in the provider variable. The chi-squared test of independence was used to examine the associations among each demographic variable, trust in health information and providers, and prescription misuse, and Fisher’s exact test was used when the count was less than 5.

## 3. Results

A total of 968 American Indian individuals participated in the survey. Participants who answered less than half of the survey or did not meet the eligibility criteria (i.e., were not American Indian and over the age of 18) were not included in the final data set. After cleaning the data, the number of participants dropped to 781. Of these individuals, 628 chose to answer the question about trust in doctor information, 629 chose to answer the question about trust in religious leaders’ information, 627 chose to answer the question about trust in tribal leaders’ information, and 631 chose to answer the question about trust in family or friends’ information. It was very important to our community partners that all participants were told clearly at the start of the survey that they did not have to complete any sections that made them uncomfortable.

The mean age of participants was 42, with a standard deviation of 16.8. The majority of the participants were American Indian alone (90.7%) rather than in combination with other races or ethnicities. Seventy-nine percent of the participants grew up on a reservation or tribal trust land (79.2%). The sample had more women (60.8%) than men (39.2%). Full demographic information is presented in Table 1.

Less than 50% of respondents answered from where they most recently received health information. Among those who responded, 28.7% answered doctors or health care providers, 21.5% cited the internet, and 17.5% family members. Other participants used brochures or pamphlets (8.6%), medical organizations (7.5%), books (4.4%), and other sources (11.6%), including social media, friends/co-workers, libraries, telephone information, and other sources. The majority of participants had moderate trust in health information from every source, with the highest percentage of moderate trust in information provided by family or friends (72.10%). However, 36.46% of participants have high trust in information provided by doctors, followed by trust in information from spiritual leaders (27.66%). Participants have less trust in information provided by tribal leaders, with only 16.75% having high trust in information from this group.

Demographic variables were examined for significant differences based on trust in providers’ information, defined as whether or not participants trusted information about health and medical topics from their providers (see Table 1). Overall, 229 participants answered that they had high trust in the information provided by their doctors (29.3%), 371 participants answered that they had moderate trust (47.5%), and 28 answered that they had no trust (3.6%). Trust in provider information differed significantly by gender. Women were more likely to have trust in information from their providers than men (*p* = 0.002). This suggests that men in this community are at higher risk for medical mistrust, potentially due to different patterns of healthcare utilization or hospital experiences with the healthcare system. Trust in doctors’ information also differed significantly by race and ethnicity. Participants who identified as American Indian alone were more likely to have moderate trust in information from their doctors than those who identified as American Indian in combination with another race or ethnicity, who had higher trust (*p* = 0.040).

Trust levels also differed significantly by whether or not someone had health insurance (*p* = <0.0001). Participants with private insurance had higher trust in information from doctors, followed by those with Medicare, Medicaid, or Veterans Affairs/TRICARE, while those with no insurance reported the lowest levels of trust (only 23.4% high trust) compared to those with private or public insurance. Therefore, uninsured individuals represent a high-risk group for mistrust, likely driven by structural barriers to care and a lack of consistent healthcare visits, thus increasing the risk of sharing medications or not finishing prescriptions due to cost and or access. Individuals who received most of their health care at a private clinic or hospital had higher trust than those receiving care from tribal health clinics or the Indian Health Service. Individuals who received their health care from a traditional healer had no trust in information from doctors (*p* = <0.0001).

Participants who had one person they considered to be their personal provider had higher trust in information from a doctor, followed by those participants with more than one personal provider. Those without a personal provider were the most likely to have no trust (Fisher’s exact test = 27.486; *p* = <0.001). Thus, having no personal doctor reduces the likelihood of receiving culturally competent care, leading to higher prescription drug misuse. Trust in information also differed significantly by the last time a participant had visited a provider for a routine check-up. Participants who had never seen a provider for a routine check-up tended to have no trust, while those who had visited the doctor within the last 12 months had higher trust (Fisher’s exact test = 27.486; *p* = <0.001).

Variables related to health status were examined for associations with trust in information from doctors, including self-reported overall health status, Native spiritual health, anxiety, depression, perceived stress, illicit drug use, and prescription medication misuse (see Table 2). Native spiritual health (*p* = 0.661), anxiety (*p* = 0.535), depression (*p* = 0.719), and perceived stress (*p* = 0.078) were not associated with trust in information about health and medical topics from doctors.

Self-reported overall health status was associated with trust in information about health and medical topics from doctors (*p* = <0.001). Trust levels differ between individuals who report misusing prescription medications and those who do not, indicating a statistically significant association. Participants who answered “no” to the question of misusing prescription drugs show higher trust (40.00%) in information about health and medical topics from doctors compared to those who have misused prescription medications (30.00%) (*p* = 0.032).

## 4. Discussion

This study explored trust in health information and, by extension, providers by asking participants if they trusted information about health and medical topics from their providers. To reduce response bias, we did not want to ask directly if they trusted medical providers themselves. Thus, these results should be interpreted carefully. The demographic variables and variables related to the overall health and mental health of participants were evaluated against trust in doctors. We also report on trust in health information provided by other sources, such as spiritual leaders, tribal leaders, and family or friends.

This cross-sectional study provides insights into a topic that has not been fully explored among American Indians. These results show a difference in trust between those who are American Indian alone and those who are American Indian in combination with other races or ethnicities, with those individuals claiming only American Indian heritage having lower trust in information about health and medical issues provided by doctors than those with mixed heritage. As noted by Pacheco et al. [6], historical grievances (including forced sterilization and unethical research) have fostered a legacy of mistrust that is often more pronounced in those with closer ties to excluded communities. In addition, negative encounters with insensitive non-Native healthcare providers continue to foster mistrust, increasing dissatisfaction among American Indian and Alaska Native patients [33,34,35]. This study reinforces the theory that medical mistrust is a multigenerational phenomenon, where negative experiences are passed down, regardless of an individual’s personal clinical history.

Healthcare distrust among all people of color, including American Indians and Alaska Natives, is also correlated with historical trauma. Canales et al. [33] identified medical mistrust as a major barrier to care for the American Indians and Alaska Natives. For some, the breaches in trust were personal, while for others, mistrust was a result of negative health care experiences of family members, some of whose stories had been passed on from generation to generation. Historical trauma and racial discrimination associated with the healthcare system distrust in this population have been found to result in less favorable attitudes toward seeking mental health services specifically [33].

However, these results also show that other demographic characteristics may positively influence participants’ trust in the medical community. For example, being female, having a person you think of as your personal doctor or personal healthcare provider, and seeing a doctor regularly are positively related to higher trust in health information provided by a doctor. Studies reviewing trends in seeing a medical provider show that women have more contact with primary care providers, and the association between relational continuity and trust in providers, visiting the same provider or team of providers, improves the relationship between providers and patients. The strongest correlation between having a personal doctor and higher trust (*p* < 0.0001) suggests that relational continuity may mitigate some effects of historical mistrust. This mirrors findings in the general US population, where visiting the same provider improves patient satisfaction and adherence [36,37].

Factors associated with greater access to a healthcare provider, such as health insurance and the location of the primary care provider, are also shown to be significantly associated with trust in doctors. For example, those with private insurance or Medicare, Medicaid, or Tricare had higher trust in health information provided by doctors. These individuals also likely have increased continuity of care, which increases their trust in doctors, as discussed previously. It is likely that all of these factors combined increase trust.

Other factors that were significantly associated with trust in health information provided by doctors included self-reported excellent and good health, illicit drug use, and prescription drug misuse. Previous research conducted with the United States general population shows that individuals with low institutional trust in the health-care system have poor self-perceived health [37]. Our study confirms this research among American Indians in the Plains, with participants self-reporting excellent and good health having higher trust in health information provided by doctors. Though we did not ask about their trust in health care institutions, it is likely that individuals who trust the health information provided by their doctors also trust the healthcare system and vice versa.

Participants in our study who reported having a primary care provider in a private clinic or a hospital had higher trust in the health information provided by their doctors. However, for those whose primary care provider was located in the Indian Health Service (IHS) or a tribal clinic, their trust in the information provided by their doctors was low. This may be explained by a lack of continuity of care in these locations. Both the IHS and tribal clinics tend to have significant turnover in personnel, leading to patients seeing different providers every time they seek care. In addition, patients often see different types of providers each time, including physicians, physician assistants, and nurse practitioners.

According to the U.S. Government Accountability Office report on the HIS [38], provider positions (including physicians, nurses, nurse practitioners, certified registered nurse anesthetists, certified nurse midwives, physicians’ assistants, dentists, and pharmacists) and vacancy rates are between 13% and 31% across IHS areas. Furthermore, the IHS Loan Repayment Program is an incentive to bring providers to IHS clinics. In return for payment of a portion of student loans, individuals in this program provide a two-year commitment to practice in health facilities serving American Indian and Alaska Native communities. This generates a frequent change of providers, preventing American Indian and Alaska Native patients and healthcare providers from forming the long-term relationships that would increase trust. It is possible that changing the provider variability and the type of provider impacts patient trust. This needs to be explored further, given the relationship between trust, continuity of care and health.

A notable characteristic of this study’s sample is the relatively high level of educational attainment; 25.67% of participants reported having at least a 2-year college degree, which exceeds the national average for the American Indian and Alaska Native population. This higher educational level may influence the generalizability of our findings. Research suggests that higher education is often associated with increased health literacy and more frequent navigation of complex healthcare systems, which can lead to higher levels of institutional trust [39]. Consequently, the trust levels reported in this study (29.3% high trust) might actually be higher than what would be found in a more educationally diverse AI/AN sample. Research exploring enhancing trust in providers to promote better use of prescription drugs is needed.

Limitations of this study include the time at which the data was collected (2017). While it provides a vital baseline, it may not reflect changes in trust following the COVID-19 pandemic. However, we know that healthcare delivery is still a problem among American Indians, as discussed previously. This study used a convenience sample. Thus, results may not be generalizable to all American Indian or Alaska Native people, who have diverse cultural and geographical contexts. All data, including drug misuse and trust, were self-reported. To reduce bias, trust was measured regarding information rather than the provider directly, which requires careful interpretation. An in-depth qualitative study is needed to help us better understand those reasons.

## 5. Conclusions

While historical trauma likely explains some mistrust in healthcare providers among American Indians, this study shows that other factors may positively affect trust. Having one person to consider as a personal primary care provider, seeing that person regularly, having health insurance (supporting relationships with individual providers), and other factors that promote continuity of care increase the trust American Indians have in doctors and healthcare providers. It is possible that we can counter some of the effects of historical trauma on poor health through intervening on these factors related to continuity of care. In addition, the positive association we found between trust in providers and appropriate use of prescription drugs suggests that we may be able to impact prescription drug misuse simultaneously. Further research is needed in this area to more fully understand the complex relationships among trust, continuity of care, health status, and prescription drug misuse.

## Figures and Tables

**Table 1:** Demographic Characteristics of Participants by Trust in Provider Information.

Demographic Variable		Total N (%)	Trust in Provider Information			*p*-Value
			High N (%)	Moderate N (%)	No N (%)	
Gender						0.002
	Female	380 (60.51)	140 (22.29)	232 (36.94)	8 (1.27)	
	Male	248 (39.49)	89 (14.17)	139 (22.13)	20 (3.18)	
Education						0.443 ^a^
	Some high school or below	61 (12.63)	18 (29.51)	41 (67.21)	2 (3.28)	
	High school graduate, technical school or some college	297 (61.49)	108 (36.36)	170 (57.24)	19 (6.40)	
	2- or 4-year college degree or graduate degree	124 (25.67)	51 (41.13)	69 (55.65)	4 (3.23)	
Race/Ethnicity						0.040 ^a^
	American Indian alone	569 (91.04)	198 (34.80)	344 (60.46)	27 (4.75)	
	American Indian in combination with another	56 (8.96)	29 (51.79)	26 (46.43)	1 (1.79)	
Location Where Participant Grew Up						0.728
	Reservation or Tribal Trust Land	494 (79.42)	179 (36.23)	294 (59.51)	21 (4.25)	
	Other	128 (20.58)	49 (38.28)	72 (56.25)	7 (5.47)	
Relationship Status						0.334
	Married or Living with a Partner	268 (44.89)	99 (36.94)	161 (60.07)	8 (2.99)	
	Not Married or Living with a Partner	329 (55.11)	118 (35.87)	193 (58.66)	18 (5.47)	
Number of Children in Household						0.013 ^a^
	No children	163 (36.47)	72 (44.17)	79 (48.47)	12 (7.36)	
	1–3	200 (44.74)	74 (37.0)	123 (61.50)	3 (1.50)	
	4+	84 (18.79)	29 (34.52)	51 (60.71)	4 (4.76)	
Employment Status						0.053
	Employed	214 (46.62)	90 (42.06)	117 (54.67)	7 (3.27)	
	Unemployed or Retired	245 (53.38)	79 (32.24)	151 (61.63)	15 (6.12)	
Health Insurance						<0.0001 ^a^
	No insurance	142 (29.46)	33 (23.24)	95 (66.90)	14 (9.86)	
	Tribal insurance or Indian Health Service	123 (25.52)	38 (30.89)	82 (66.67)	3 (2.44)	
	Private insurance	74 (15.35)	41 (55.41)	33 (44.59)	0 (0.00)	
	Medicare, Medicaid, or Veterans Affairs/TRICARE	143 (29.67)	65 (45.45)	71 (49.65)	7 (4.90)	
Location of Primary Medical Care						<0.0001 ^a^
	Indian Health Service	317 (74.07)	110 (34.70)	192 (60.57)	15 (4.73)	
	Tribal clinic	45 (10.51)	17 (37.78)	26 (57.78)	2 (4.44)	
	Private clinic or hospital	60 (14.02)	37 (61.67)	21 (35.00)	2 (3.33)	
	Traditional healer	6 (1.140)	0 (0.00)	3 (50.00)	3 (50.00)	
Do you have one person you think of as your personal doctor or health care provider?						<0.0001 ^a^
	Yes, one	125 (25.83)	70 (56.00)	53 (42.40)	2 (1.60)	
	Yes, more than one	114 (23.55)	50 (43.86)	59 (51.75)	5 (4.39)	
	No	245 (50.62)	57 (23.27)	170 (69.39)	18 (7.35)	
Type of personal provider seen regularly						<0.0001 ^a^
	Doctor	244 (59.66)	111 (45.49)	125 (51.23)	8 (3.28)	
	Nurse Practitioner or Physician’s Assistant	17 (4.16)	3 (17.65)	10 (58.82)	4 (23.53)	
	Traditional healer	124 (30.32)	35 (28.23)	82 (66.13)	7 (5.65)	
	Other	24 (5.87)	4 (16.67)	16 (66.67)	4 (16.67)	
Was there a time in the last 12 months when you needed to see a doctor but could not because of cost?						0.066
	Yes	128 (26.67)	38 (29.69)	80 (62.50)	10 (7.81)	
	No	352 (73.33)	139 (39.49)	198 (56.25)	15 (4.26)	
Time since last routine check-up						<0.001
	Within the past 12 months	440 (70.40)	170 (38.64)	261 (59.32)	9 (2.05)	
	More than 1 year, but less than 2 years ago	87 (13.92)	23 (26.44)	59 (67.82)	5 (5.75)	
	More than 2 years, but less than 5 years ago	34 (5.44)	10 (29.41)	22 (64.71)	2 (5.88)	
	5 or more years ago	33 (5.82)	16 (48.48)	14 (42.42)	3 (9.09)	
	Never	31 (4.96)	9 (29.03)	13 (41.94)	9 (29.03)	

^a^ When the expected count is less than 5, Fisher’s exact test was conducted. *p* < 0.05; *p* < 0.01; percentages do not always total 100% due to rounding; participants could choose to answer or not answer individual questions; thus, N is different for different variables.

**Table 2:** Health of Participants and Trust in Provider Information.

Health Variable		Total N (%)	Trust in Provider Information			*p*-Value
			High N (%)	Mod N (%)	No N (%)	
Would you say that in general your health is _______?						<0.001
	Excellent	94 (15.02)	44 (46.81)	39 (41.19)	11 (11.70)	
	Very good	138 (22.04)	53 (38.41)	82(59.42)	3 (2.17)	
	Good	257 (41.05)	90 (35.02)	163 (63.42)	4 (1.56)	
	Fair	129 (20.61)	40 (31.01)	80 (62.02)	9 (6.98)	
	Poor	8 (1.28)	2 (25.00)	5 (62.50)	1 (12.50)	
Native Spirituality Scale						0.661
	High	269 (45.2)	103 (38.29)	152 (56.51)	14 (5.20)	
	Moderate	147 (24.87)	55 (37.41)	84 (57.14)	8 (5.44)	
	Low	175 (29.61)	62 (35.43)	108 (61.71)	5 (2.86)	
Beck Anxiety Inventory						0.535 ^a^
	Concerning anxiety	102 (22.30)	40 (39.20)	59 (57.80)	3 (2.90)	
	Moderate anxiety	206 (45.10)	78 (37.90)	119 (57.80)	9 (4.40)	
	Low anxiety	149 (32.60)	50 (33.60)	88 (59.10)	11 (7.40)	
CESD-10						0.719 ^a^
	Depressed	113 (23.3)	42 (37.20)	67 (59.30)	4 (3.50)	
	Not depressed	372 (76.7)	133 (35.80)	218 (58.60)	21 (5.60)	
Perceived Stress Scale						0.073 ^a^
	High perceived stress	25 (9.90)	10 (40.00)	15 (60.00)	0 (0.00)	
	Moderate perceived stress	143 (56.70)	48 (33.60)	88 (61.50)	7 (4.90)	
	Low perceived stress	84 (33.30)	28 (33.30)	44 (52.40)	12 (14.30)	
Illicit Drug Use						0.075
	Yes	363 (60.90)	138 (38.00)	203 (55.90)	22 (6.10)	
	No	233 (49.40)	81 (34.80)	146 (62.70)	6 (2.60)	
Prescription Drug Misuse						0.032
	Yes	200 (43.00)	60 (30.00)	132 (66.00)	8 (4.00)	
	No	265 (57.00)	106 (40.00)	143 (54.00)	16 (6.00)	
Finishing Course of Antibiotics						0.193
	Always finish	264 (57.10)	92 (34.80)	154 (58.30)	18 (6.80)	
	Do not always finish	198 (42.90)	74 (37.40)	118 (59.60)	6 (3.00)	

^a^ When the expected count is less than 5, Fisher’s exact test was conducted *p* < 0.05; *p* < 0.01; percentages do not always total 100% due to rounding; participants could choose to answer or not answer individual questions, thus N is different for different variables.

## Data Availability

The data presented in this study are available on request from the corresponding author due to privacy reasons.
